# Positive association of familial longevity with the moderate-high HDL-C concentration in Bama Aging Study

**DOI:** 10.18632/aging.101663

**Published:** 2018-11-28

**Authors:** Jian Wang, Liwei Shi, Yunfeng Zou, Jiexia Tang, Jiansheng Cai, Yi Wei, Jian Qin, Zhiyong Zhang

**Affiliations:** 1 Department of Occupational and Environmental Health, School of Public Health, Guangxi Medical University, Nanning, China; 2 Guangxi Colleges and Universities Key Laboratory of Prevention and Control of Highly Prevalent Diseases, Guangxi Medical University, Nanning, China; 3 Insitute of Vaccine Clinical Research, Guangxi Center for Disease Prevention and Control, Nanning, China; 4 Department of Toxicology, School of Public Health, Guangxi Medical University, Nanning, China; 5 Guilin Medical University, Guilin, China; * Equal contribution

**Keywords:** familial longevity, offspring, HDL-C, APOE

## Abstract

Familial longevity is characterized by beneficial metabolic phenotype in lipid metabolism and *APOE* genetic variation. Although effects of lipid metabolism and the genetic basis for human longevity remain largely unclear, the contribution of high-density lipoprotein cholesterol (HDL-C) and *APOE* ε2 allele has been repeatedly demonstrated. This study was designed to determine whether ApoE isoforms and HDL-C levels marked the familial longevity status in an offspring cohort with the age range of 20-89 years old and subsequently to explore the correlation between these two markers and the aging. In the Bama Aging Study (BAS), we recruited 312 offspring from longevity historical families and 298 controls from non-longevity historical families. Information on *APOE* genotype frequencies, lipid levels, and population characteristics were recorded. No evidence was found to support the association of *APOE* genotypes with HDL-C and age. HDL-C was significantly higher in longevity group (p < 0.0001). Scatter plot showed a moderately strong linear relationship between the HDL-C level and age in longevity group (r = 0.213, p < 0.001). We conclude that the variation of the *APOE* gene may not influence familial longevity status at a certain age but the moderate-high HDL-C level contributes to the familial longevity in Bama.

## Introduction

The association between high-density lipoprotein cholesterol (HDL-C) and longevity was initially suggested by Glueck et al in 1975 [1], and this was followed by the Long Life Family Study (LLFS) in US, which showed that the proband and offspring had higher HDL-C levels and lower cardiovascular risk factors as compared to normal individuals [2]. In addition, HDL-C as an inverse predictor of cardiovascular disease (CVD) has been appreciated when the patients are over 50 years old. Low HDL-C has been found to be either highly prevalent in patients with CVD or to increase the risk of myocardial infarction (MI), which is independent from other CVD risk factors [3–5]. High level of HDL-C as a negative risk factor might be used to counteract the presence of other traditional CVD risk factors [6,7]. But it still remains controversial whether the high HDL-C is reliable indicator of longevity.

For decades, it has been widely accepted that the human exceptional longevity is a complex interaction between environmental and multiple genetic factors. Apolipoprotein E (ApoE), as a longevity-associated gene [8], is a major cholesterol carrier mediates lipid transport. The *APOE* gene is polymorphic, and the vast majority of population studies have focused on the three most common alleles (ε2, ε3 and ε4) that result in six possible genotypes [9]. Both reduction in the frequency of the ε4 allele and increase in the frequency of the ε2 allele contribute to longevity [10,11]. Some studies, however, did not show a significant association between ε2 and exceptional longevity [12–14]. Interestingly, data also support plasma cholesterol and HDL-C levels were lowest in those with ε2 allele, compared to those of ε3 and ε4 alleles [15]. *APOE* ε4 is associated with higher plasma cholesterol levels [16]. Despite sustained interest, the impact of plasma HDL-C levels and *APOE* gene on human longevity remains unclear.

Bama County, located in the Hongshuihe River Basin of Guangxi Province, is well known as a longevity area around the world, as its lower genetic diversity background [17,18]. It is a good model for long life study. Thus, in this study, we intended to investigate the correlation between *APOE* genotypes, lipid profiles and human longevity using data from the Bama Aging Study.

## RESULTS

### Participant characteristics at baseline

Participant demographic and *APOE* genotype are listed in Table 1. The sample included 610 adults (294 female) with an average age of 64.4 years. There were no statistical differences in the distribution of BMI, alcohol intake and current smoking status among the subjects with different *APOE* genotype (p > 0.05). However, after adjusted for age, sex, BMI, alcohol intake and smoking status, levels of SBP, total cholesterol (TC) and low-density lipoprotein cholesterol (LDL-C) were significantly different among *APOE* ε2 genotype group (p = 0.003, p = 0.004, p = 0.013, respectively, Table 1). TC and LDL-C levels were lower in ε2 carriers than those of ε2 non-carriers. Interestingly, SBP level in the ε2 homozygote group was highest. Triglycerides (TG) level and C-index 1 and 2 were significantly different among *APOE* ε4 genotypes (p = 0.048, p < 0.0001, p = 0.005, respectively), and ε4 homozygote carriers had the highest levels (Table 1).

**Table 1:** Participant characteristics at baseline in different *APOE* genotype groups.

Variables	All participants	ε 2 noncarrier	ε 2 homozygote	ε 2 heterozygote	p-value	ε 4 noncarrier	ε 4 homozygote	ε 4 heterozygote	p-value
	(n = 610)	(n = 521)	(n = 5)	(n = 66)		(n = 480)	(n = 6)	(n = 106)	
Mean age, y	64.4 (18.1)	64.5 (18.2)	73.4 (4.2)	64.2 (18.0)	0.731	64.0 (18.1)	63.7 (15.2)	67.1 (18.1)	0.377
Female, n (%)	294 (48.2)	250 (48.0)	4 (80.0)	34 (51.5)	0.457	231 (48.1)	3 (50)	54 (51.0)	0.982
BMI	20.6 (2.8)	20.7 (2.8)	20.4 (2.1)	20.5 (2.4)	0.725	20.7 (2.8)	21.6 (3.2)	20.5 (2.9)	0.589
Alcohol intake, n (%)	281 (46.1)	245 (47.0)	1 (20.0)	31 (47.0)	0.483	228 (47.5)	3 (50)	46 (43.4)	0.736
Current smoking, n (%)	247 (40.5)	212 (40.7)	1 (20.0)	28 (42.4)	0.616	190 (39.6)	4 (66.7)	47 (44.3)	0.286
SBP, mmHg	132.7 (21.7)	132.7 (21.6)	166.2 (22.9)	130.5 (20.5)	0.003*	132.3 (22.2)	131.7 (14.8)	134.9 (20.2)	0.865
DBP, mmHg	79.3 (11.2)	79.2 (11.2)	84.2 (15.0)	78.4 (11.4)	0.551	79.1 (11.4)	79.7 (5.7)	79.0 (10.7)	0.963
Glucose, mmol/L	5.4 (1.5)	5.4 (1.5)	6.9 (1.4)	5.4 (1.6)	0.095	5.4 (1.4)	6.3 (1.9)	5.6 (1.9)	0.096
Total Cholesterol, mmol/L	4.8 (0.9)	4.8 (0.9)	3.8 (1.0)	4.6 (0.8)	0.004*	4.8 (0.9)	5.6 (0.8)	4.8 (1.0)	0.138
HDL-Cholesterol, mmol/L	1.3 (0.7)	1.4 (0.7)	1.6 (1.4)	1.3 (0.6)	0.560	1.4 (0.7)	1.3 (0.9)	1.3 (0.6)	0.688
LDL-Cholesterol, mmol/L	2.8 (0.8)	2.8 (0.8)	2.0 (0.8)	2.7 (0.7)	0.013*	2.8 (0.8)	3.1 (1.4)	2.8 (0.8)	0.785
Triglycerides, mmol/L	1.6 (1.4)	1.6 (1.4)	1.5 (0.3)	1.7 (1.3)	0.835	1.6 (1.3)	3.0 (2.5)	1.6 (1.3)	0.048*
C-index 1	4.1 (2.3)	4.1 (2.3)	3.5 (1.8)	4.0 (1.1)	0.838	4.0 (1.3)	7.5 (7.9)	4.4 (4.0)	<0.0001*
C-index 2	2.4 (1.4)	2.4 (1.4)	2.0 (1.1)	2.3 (0.7)	0.655	2.3 (1.0)	4.1 (4.7)	2.5 (2.1)	0.005*

Note: Variables were compared using statistically appropriate methods and *APOE* ε2/ε4 heterozygotes were omitted from the analysis.

Abbreviations: BMI, body mass index; SBP, systolic blood pressure; DBP, diastolic blood pressure; HDL, high-density lipoproteins; LDL, low-density lipoproteins; C-index 1, castelli’s index 1; C-index 2, castelli’s index 2. Data presented as the mean (SD) unless indicated otherwise.

*ANCOVA comparing *APOE* genotype groups adjusted for age, sex, BMI, drink and smoking status.

*Statistically Significant.

### Comparisons of study variables between longevity and non-longevity families

We compared study variables such as SBP, DBP, Glucose and lipid profiles using binary logistic regression model. Table 2 displays the statistical differences in analyses adjusted for age, sex, BMI, alcohol intake, smoking status and *APOE* genotypes. Interestingly, SBP and HDL-C levels were significantly higher in longevity families than those of non-longevity families (p < 0.0001). While LDL-C, C-index 1 and 2 levels were significantly increased in non-longevity families (p = 0.008, p < 0.0001, p < 0.0001, respectively), as compared with subjects from longevity families. There was no statistical difference in TG level when comparing subjects between the longevity and non-longevity families (p = 0.844). We also characterized the association between SBP, DBP, Glucose, lipid profiles and participant baseline characteristics by using linear regression model (Supplementary Table S1). The linear effects of age on DBP and HDL-C levels were observed in both families (p < 0.05).

**Table 2:** Comparison of influencing factors of aging and health between longevity and non-longevity groups.

Variables	Longevity family	Non-longevity family	p-value
	(n=304)	(n=288)	
Mean age, y	66.7 (18.1)	62.1 (17.9)	0.0018*
Female, n (%)	139 (44.6)	155 (52.0)	0.075
BMI	20.8 (2.8)	20.5 (2.8)	0.366
Alcohol intake, n (%)	119 (38.1)	162 (54.4)	<0.0001*
Current smoking, n (%)	107 (34.3)	140 (47.0)	0.0017*
APOE2+, n (%)	36 (11.5)	35 (11.7)	0.998
APOE4+, n (%)	54 (17.3)	58 (19.5)	0.531
SBP, mmHg	137.6 (21.9)	127.7 (20.3)	<0.0001*
DBP, mmHg	80.5 (10.9)	80.0 (11.3)	0.060
Glucose, mmol/L	5.5 (1.5)	5.3 (1.5)	0.281
TC, mmol/L	4.9 (0.9)	4.7 (0.9)	0.082
HDL-C, mmol/L	1.6 (0.8)	1.1 (0.3)	<0.0001*
LDL-C, mmol/L	2.7 (0.7)	2.9 (0.8)	0.008*
TG, mmol/L	1.7 (1.5)	1.6 (1.3)	0.844
C-index 1	3.65 (1.61)	4.56 (2.72)	<0.0001*
C-index 2	2.05 (1.09)	2.75 (1.49)	<0.0001*

Note: Parameters were analyzed using binary logistic regression model and *APOE* ε2/ε4 heterozygotes were omitted from the analysis. Abbreviations: APOE2+, *APOE* ε2 carriers; APOE4+, *APOE* ε4 carriers. Data presented as the mean (SD) unless indicated otherwise. *Statistically Significant.

### Comparisons of *APOE* genotype frequencies and HDL-C levels by age subgroups in longevity and non-longevity families

There was no statistically significant difference in *APOE* genotype frequencies between young subjects and old subjects in longevity family. The distribution of *APOE* ε4 noncarriers and ε4 heterozygote carriers was significant difference between young subjects and old subjects in non-longevity family (p = 0.005, p = 0.001, respectively). Comparing subjects age ≥ 60 years with younger subjects, the HDL-C level was significantly higher in the older age group in longevity family and higher in younger age group in non-longevity family (p < 0.001, p = 0.009, respectively). (Table 3)

**Table 3:** *APOE* genotype frequencies and HDL-C levels by age subgroups in longevity and non-longevity families.

Variables	Longevity family		p-value	Non-longevity family		p-value
	Age < 60 (n = 129)	Age ≥ 60 (n = 175)		Age < 60 (n = 143)	Age ≥ 60 (n = 145)	
*APOE* genotype, (n, %)						
ε2 noncarrier	117 (90.7)	151 (86.3)	0.283	125 (87.4)	128 (88.3)	0.858
ε2 homozygote	0 (0)	3 (1.7)	–	0 (0)	2 (1.4)	–
ε2 heterozygote	12 (9.3)	21 (12.0)	0.576	18 (12.6)	15 (10.3)	0.583
ε4 noncarrier	108 (83.7)	142 (81.2)	0.649	124 (86.7)	106 (73.1)	0.005*
ε4 homozygote	0 (0)	1 (0.5)	–	4 (2.8)	1 (0.7)	0.212
ε4 heterozygote	21 (16.3)	32 (18.3)	0.760	15 (10.5)	38 (26.2)	< 0.001*
HDL-C, mmol/L	1.378 (0.79)	1.734 (0.81)	< 0.001*	1.158 (0.38)	1.060 (0.22)	0.009*

Note: Mann Whitney U-tests and Fisher’s exact tests were used to compare the difference between age subgroups in longevity and non-longevity families. Participants carrying the *APOE* ε2/ε4 isoform were excluded. Data presented as the mean (SD) unless indicated otherwise. *Statistically Significant.

### Correlations of age with SBP, DBP, Glucose and lipid profiles

**Figure 1:**
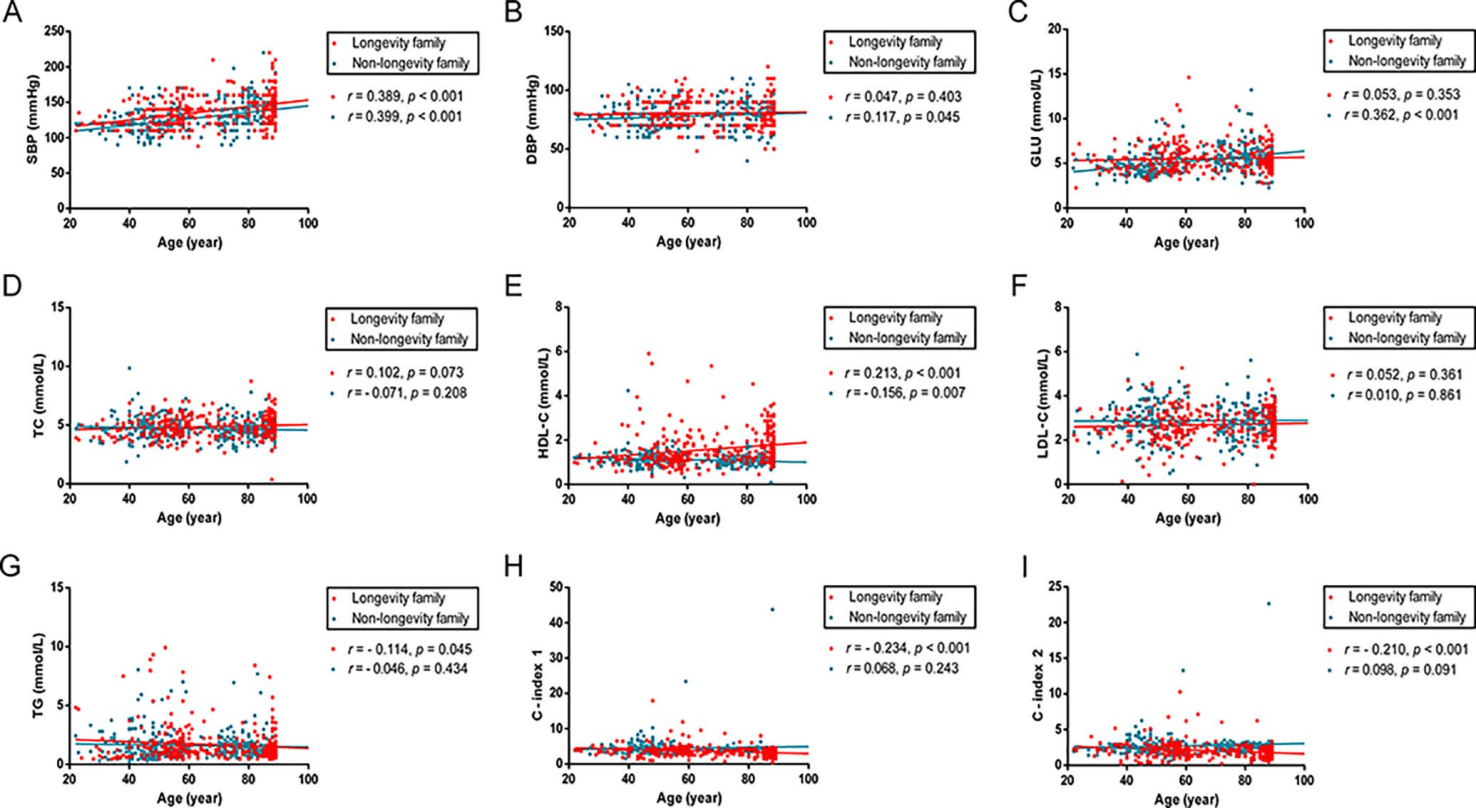
**Scatter plots and linear fit line of participants’ age versus SBP, DBP, Glucose and plasma lipid profiles.** Participants spanned 20 - 89 years of age. Each dot represents an individual in longevity family (red) and non-longevity family (blue). (**A**) SBP levels are positively correlated with age both in longevity and non-longevity families. (**B, C**) DBP and GLU levels are positively correlated with age in non-longevity family, but no statistically significant difference in longevity family. (**D, F**) TC and LDL levels are not statistically significantly correlated with age in both families. (**E**) HDL levels are positively correlated with age in longevity family and negatively correlated with age in non-longevity family. (**G, H, I**) TG, C – index 1 and C – index 2 levels are negatively correlated with age in longevity family, but no statistically significant difference in non-longevity family. Correlation coefficient (r) and P-value were acquired by Pearson correlation test. For these analyses, 18 participants carrying the ε2/ε4 isoform were excluded. Abbreviations: SBP, systolic blood pressure; DBP, diastolic blood pressure; GLU, glucose; TC, total cholesterol; HDL, high-density lipoproteins; LDL, low-density lipoproteins; TG, triglycerides; C-index 1, castelli’s index 1; C-index 2, castelli’s index 2.

The correlations of age with SBP, DBP, Glucose and lipid profiles were examined using Pearson test (Fig 1). In longevity families, age was positively correlated with SBP and levels of HDL-C (r = 0.389, p < 0.001; r = 0.213, p < 0.001; respectively) and negatively associated with TG levels, C-index 1 and 2 (r = -0.114, p = 0.045; r = -0.234, p < 0.001; r = -0.210, p < 0.001; respectively). In non-longevity families, age was positively associated with SBP, DBP and Glucose (r = 0.399, p < 0.001; r = 0.117, p = 0.045; r = 0.362, p < 0.001; respectively) and negatively associated with HDL-C levels (r = -0.156, p = 0.007).

## DISCUSSION

In the current study, we found that TC and LDL-C levels were lower in *APOE* ε2 carriers compared with ε2 non-carriers. TG, C-index 1 and 2 levels were significantly different between subjects with different among *APOE* ε4 genotypes and ε4 homozygote carriers with the highest levels. Adjustment for common confounding conditions, including age, sex, BMI, alcohol intake and smoking status did not change the pattern of results.

Previous studies have reported that ApoE is a multifunctional protein that plays a key role in the metabolism of cholesterol and TG in an isoform-dependent manner [19,20]. *APOE* ε2 allele carriers have lower and those carrying ε4 allele have higher TC and LDL-C levels than people with the commonest ε3 genotype [21,22]. Lipid metabolism changes were correlated with circulating ApoE concentration and CVD risk [23]. In the population-based study, circulating ApoE was associated with SBP and DBP [24]. ε2 allele could reduce the risk of coronary disease whereas ε4 allele might slightly increase the risk [22]. Castelli indexes 1 (TC/HDL-C) and 2 (LDL-C/HDL-C) ratios are more powerful predictors of coronary heart disease (CHD) than the individual lipid used separately [25,26]. The TC/HDL-C ratio is found to present less superposition of populations [27]. TC/HDL-C ratio at 5.5 indicates moderate atherogenic risk and 3.8 is considered a more sensitive and specific parameter of cardiovascular risk than total cholesterol [26]. LDL-C/HDL-C ratio illustrates similar function to the ration of TC/HDL-C, however, LDL-C/HDL-C may have more predictive power if triglyceridemia is taken into account [28]. Increases in these ratios could indicate significantly higher risk of CVD [29]. Our results of TC, LDL-C, TG, C-index 1 and 2 levels were consistent with previously published findings. Interestingly, we found no evidence that familial longevity was mediated by *APOE* genotypes. This phenomenon has been observed in previous study, which suggests that variation at the *APOE* locus may not influence familial longevity status in middle age [30].

To investigate whether the effects of *APOE* genotype varied by age, we compared *APOE* genotype frequencies between old subjects and young subjects in either longevity family or non-longevity family. We found that the distribution of *APOE* ε4 noncarriers and ε4 heterozygote carriers was significant difference between young subjects and old subjects in non-longevity family. No difference was found in longevity family. Many investigators assume that frequencies are constant across the life span, and this is especially so when the incidence of the disease in question increases with age. It has been estimated from cross-sectional studies that the frequency distribution of the ε4 allele halves between the ages of 60 and 85 years [31]. The *APOE* ε4 allele is associated with an increased mortality risk throughout adulthood and ε4 allele is also considered a frailty gene, reducing the probability of successful aging even with a long lifespan [32]. *APOE* ε4 allele on total difference in mortality and an indication that this effect increases with age [33]. In stratified analyses for parental deaths before and after 75 years, we see a much stronger effect of ε4 on dying after 75 years, for both mothers and fathers, providing strong additional evidence for an effect on survival to >90 years [34,35]. These findings suggest that the associations between *APOE* ε4 and mortality are age dependent [36].

Our results suggest that HDL-C, as a reliable predictor, may play an important role on influencing familial longevity. The association between HDL-C and familial human longevity has been observed in multiple previous studies [2,37,38]. Epidemiologic studies have shown that high HDL-C levels have protective effects on atherosclerosis and CVD across multiple populations [39,40]. Subjects with high HDL-C levels have a lower risk of death than subjects with lower initial HDL cholesterol levels [41]. Lower HDL-C levels has been found to increase the mortality rate in both CVD and non-CVD populations [42]. These studies suggest that high levels of HDL-C may contribute to exceptional longevity, likely due in part to the reduction in CVD risk. The HDL-C concentration associated with the lowest risk of all-cause mortality was 1.9 mmol/L for men, and 2.4 mmol/L for women [43]. Patients with very low HDL-C levels (< 1.03 mmol/L) or very high (> 2.3 mmol/L) have a greater mortality rate compared with individuals with HDL-C levels that fell within intermediate ranges [42]. Both extreme high and low HDL-C concentrations are associated with high mortality [43]. In our study, the mean HDL-C level of familial longevity group is 1.6 mmol/L and 1.1mmol/L in non-longevity group. It suggests that moderate-high HDL-C level may contribute to human longevity due to influence mortality.

Although offspring of centenarians have significantly delayed onset of chronic conditions, including CHD, hypertension, diabetes, and stroke—as well as lower overall cardiovascular and cancer mortality than age-matched controls with at least one parent who died at average life expectancy [44,45]. Familial longevity influence is strongly and positively associated with physical health, mental health, and subjective well-being at old ages [46]. Non-smoking, being physically fit and having a healthy BMI were mortality risk reducers [47]. The probability of survival to age 85 years was a function of the cumulative number of midlife risk factors, including high handgrip strength and avoidance of overweight, hyperglycemia, hypertension, smoking, and excessive alcohol consumption [48]. More in-depth studies including more stringent tests of the significance and replication studies are warranted for deeper understanding of the effects of interactions between familial longevity influence and environmental exposures. Together, it is not certain whether offsprings of longevity family really can have potential to live long.

Recent studies also found that the association between HDL-C levels and cause-specific mortality outcomes appeared as a “U-shaped” rather than a linear relationship in the dose-dependent manner. Age-standardized all-cause mortality was lowest in the strata of HDL cholesterol from 1.3–1.8 mmol/L in men and from 1.6–1.8 mmol/L in women [42]. The study by Bowe et al. showed a similar U-shaped association between all-cause mortality and HDL-C in men with the lowest risk at HDL-C of 0.8–1.3 mmol/L [49]. The U-shaped curve was not expected, and the underlying mechanism of the association between high HDL-C levels and mortality is not clear. The relationship between HDL-C levels and mortality may be mediated through complex relationships involving diverse factors. HDL-C level as a modifiable risk factor [50,51] can be increased by niacin and cholesteryl ester transfer protein inhibitors which however doesn’t show improved clinical outcomes as compared with control [52,53]. In addition, high HDL-C concentration caused by genetic variant is not associated with a reduced risk of CVD [54]. This suggests that HDL-C level is a marker of poor general health and may not be an independent modifiable risk factor specifically for CVD. Interestingly, we did not find evidence of an association between ApoE isoforms and HDL-C, but we did find an association between *APOE* ε2 and TC. In contrast to prior reports, our study was conducted in Bama Zhuang area, this discrepancy may be due to the population and its genetic background, environmental and ethnic differences, sample size, or health status. The effects of *APOE* genotypes may be particularly strong in certain subgroups, such as in women [55]. In future investigation, we should focus on the quality of HDL, which cannot be reliably estimated through the simple measurement of HDL-C.

Our data showed only a part of subjects from family longevity have higher HCL-C levels, but other subjects have normal HDL-C levels (Fig 1E). Plasma high-density lipoprotein cholesterol is a quantitative trait in which complex interactions of several genes and environmental factors determine the plasma levels in the general population [56]. The genetic component, estimated to determine 50% of plasma HDL-C variability, has been investigated heavily to identify genes regulating plasma HDL-C levels [56]. Narrow sense heritability estimate (*h*^2^) for plasma HDL-C accounting for age, sex, batch, and plate was 0.51 [57]. Heritabilities of plasma HDL-C in other family-based studies have been previously reported to range from 0.42 to 0.83 [58–62]. These studies suggest that gene-by-environment interaction can mediate human plasma HDL-C levels. We also observed significant difference in HDL-C level between old subjects and young subjects in either longevity family or non-longevity family. The HDL-C level of old subjects in longevity family is higher than young subjects and lower in non-longevity family (Table 3). Consistent with our findings, another observational study comparing healthy subjects aged 85–89 years with younger subjects, the HDL-C level was higher in 92% of the elderly compared with 69% of healthy subjects aged 38–69 years, and 46% of coronary subjects aged 32–69 years [63].

Several limitations pertain in the current study. First, the sample size is relatively small due to the difficulties in collecting familial longevity samples and restriction of the analysis to Bama Zhuang people. It should be a wide range of populations of differing ethnicity and ancestry. Second, although our study data included HDL-C levels and other lipid parameters, our data do not allow for qualitative assessment of HDL-C size, composition, functional capacity, or HDL-C subclasses (i.e., HDL2, which is generally associated with improved cardiovascular outcomes) [64,65]. Third, selection bias existed amongst the longevity family member of the BAS for measurement due to inclusion criteria. More detailed work is needed to help understand reasons for the heterogeneity observed among the larger studies of ApoE and longevity. Even though these studies were focused on those aged 85 years or above. Fourth, not every offspring is enriched for longevity, because they may not inherit the favorable predisposition for longevity of their long-lived parents. And finally, results from the present study should be replicated in an independent cohort with a similar design, data collection and sample size.

In summary, although no evidence to support the association between *APOE* genotypes and offspring of long-lived families with the age range 22-89, our data clearly show a moderate increase in the HDL-C level in familial longevity group and it is positively correlated with age. Therefore, we conclude that the *APOE* genetic variation may not influence familial longevity status at a certain age but the moderate-high HDL-C level contributes to the familial longevity.

## MATERIALS AND METHODS

### Study population

Participants were recruited from the Bama Aging Study (BAS), a prospective study that aims to identify determinants of healthy aging and familial longevity. A total of 610 subjects aged from 20 to 89 years old at baseline were enrolled in our study from the Bama Zhang area. Among these 610 participants, 312 subjects were obtained from longevity historical families and 298 subjects were from non-longevity historical families. All subjects were healthy without diseases such as atherosclerosis, CVD, cancer and diabetes. The participants did not take medications that might affect plasma lipid levels (for example, statins or fibrates, beta-blockers, diuretics, or hormones). The Bama Aging Study was approved by the medical ethical committee of Guangxi Medical University. Written informed consent was obtained from all participants.

### Blood chemistry

Fasting blood samples for measurements were obtained in the morning. EDTA plasma samples were aliquoted within 6 hours and stored at -80°C. Fasting plasma samples were assayed for lipid profiles (TC, HDL-C, LDL-C, and TG) and glucose at the key laboratory of high incidence diseases prevention and control of Guangxi Universities and Colleges using commercially available kits (Roche Diagnostic, Mannheim, Germany).

### *APOE* genotyping

Our assay was based on allele-specific PCR methodology adapted to Real Time PCR monitored by TaqMan probe. Genomic DNA was extracted from whole blood samples by using the Wizard Genomic DNA Purification Kit (Promega, USA) according to manufacturer’s instruction. PCR was performed according to the standard methods. Briefly, each PCR reaction mixture (10 µL) contained the following reagents: 5 µL of TaqMan Universal PCR Master Mix, 0.25 µL of Assay-on-Demand SNP Genotyping Assay Mix (40×) (Applied Biosystems, USA), 1 µL of genomic DNA and 3.75 µL of ddiH_2_O. The PCR cycling conditions were setup with initial denaturation at 95°C for 10 min followed by 40 cycles with denaturation at 92°C for 15 sec, annealing at 60°C for 1 min, extension at 72°C for 60 sec. The fluorescence signals were collected during the annealing/extension step and performed by SDS 2.3 software (Applied Biosystems, USA).

### Statistical analysis

Demographic and baseline characteristics within each *APOE* genotype group were assessed using summary statistics. Comparisons between *APOE* genotype groups were performed using ANCOVA tests, Dunn’s multiple comparison’s tests and χ^2^ tests for categorical variables. To assess family-specific difference of participant health information, comparisons were performed using binary regression tests, χ^2^ tests and Mann Whitney U-tests. General Linear models were used to examine the correlations between lipid profiles and factors influencing longevity. Pearson correlation test was used to summarize the degree of correlation between age and SBP, DBP, Glucose and lipid profiles. *APOE* ε2/ε4 heterozygote carriers were omitted. Statistical analyses were performed using SPSS 16.0 or Prism 6.0. P-values < 0.05 were considered significant.

## SUPPLEMENTARY MATERIAL

Supplementary Table S1
