# Supplementary material for: Positive association of familial longevity with the moderate-high HDL-C concentration in Bama Aging Study
Source: Aging (Albany NY). 2018 Nov 28;10(11):3528–40. doi: 10.18632/aging.101663 (PMC6286851; doi:10.18632/aging.101663)
Supplement: Supplementary Table S1 [file aging-10-101663-s001.docx]

**Supplementary Table S1. Association between lipid profiles and factors influencing longevity.**

|  | Model | Longevity family | | | | Non-longevity family | | | |
| --- | --- | --- | --- | --- | --- | --- | --- | --- | --- |
|  |  | Coefficient | Lower 95% | Upper 95% | *p*-value | Coefficient | Lower 95% | Upper 95% | *p*-value |
| SBP | Constant | 99.461 | 72.298 | 126.625 | 0.000 | 84.066 | 55.257 | 112.875 | 0.000 |
|  | Age | 0.483 | 0.336 | 0.631 | <0.0001* | -1.594 | -5.864 | 2.676 | 0.463 |
|  | Sex | -1.441 | -7.099 | 4.218 | 0.617 | 0.491 | 0.341 | 0.641 | <0.0001* |
|  | BMI | 0.559 | -0.286 | 1.404 | 0.194 | 1.266 | 0.371 | 2.162 | 0.006* |
|  | Alcohol | 0.105 | -5.304 | 5.515 | 0.969 | -7.004 | -11.875 | -2.134 | 0.005* |
|  | Smoking | -2.608 | -8.296 | 3.080 | 0.368 | -0.511 | -5.534 | 4.511 | 0.841 |
|  | APOE2+ | -1.851 | -9.078 | 5.375 | 0.615 | 3.581 | -2.987 | 10.149 | 0.284 |
|  | APOE4+ | -0.420 | -6.535 | 5.695 | 0.893 | 3.595 | -1.820 | 9.011 | 0.192 |
| DBP | Constant | 77.419 | 62.868 | 91.971 | 0.000 | 63.431 | 45.807 | 81.055 | 0.000 |
|  | Age | 0.080 | 0.001 | 0.159 | 0.047* | 0.112 | 0.020 | 0.204 | 0.017* |
|  | Sex | -3.431 | -6.463 | -0.400 | 0.027* | -1.618 | -4.230 | 0.994 | 0.224 |
|  | BMI | 0.101 | -0.352 | 0.553 | 0.662 | 0.728 | 0.180 | 1.276 | 0.009* |
|  | Alcohol | 0.708 | -2.190 | 3.606 | 0.631 | -3.147 | -6.126 | -0.167 | 0.039* |
|  | Smoking | 0.013 | -3.034 | 3.061 | 0.993 | -0.135 | -3.208 | 2.937 | 0.931 |
|  | APOE2+ | -1.287 | -5.158 | 2.585 | 0.514 | 0.478 | -3.540 | 4.497 | 0.815 |
|  | APOE4+ | -1.226 | -4.502 | 2.050 | 0.462 | 0.494 | -2.819 | 3.807 | 0.769 |
| Glucose | Constant | 3.916 | 1.925 | 5.908 | 0.000 | 3.218 | 1.023 | 5.413 | 0.004 |
|  | Age | 0.008 | -0.003 | 0.019 | 0.155 | 0.031 | 0.020 | 0.042 | <0.0001* |
|  | Sex | -0.251 | -0.666 | 0.164 | 0.234 | -0.161 | -0.486 | 0.164 | 0.331 |
|  | BMI | 0.080 | 0.018 | 0.142 | 0.012* | 0.015 | -0.053 | 0.083 | 0.663 |
|  | Alcohol | 0.110 | -0.286 | 0.507 | 0.584 | -0.139 | -0.510 | 0.232 | 0.461 |
|  | Smoking | -0.315 | -0.732 | 0.102 | 0.138 | 0.121 | -0.262 | 0.504 | 0.535 |
|  | APOE2+ | 0.170 | -0.360 | 0.699 | 0.529 | 0.331 | -0.169 | 0.832 | 0.193 |
|  | APOE4+ | 0.304 | -0.145 | 0.752 | 0.184 | 0.234 | -0.179 | 0.647 | 0.265 |
| TC | Constant | 3.751 | 2.497 | 5.004 | 0.000 | 2.629 | 1.155 | 4.103 | 0.001 |
|  | Age | 0.007 | 0.000 | 0.014 | 0.043* | 0.002 | -0.006 | 0.010 | 0.605 |
|  | Sex | 0.097 | -0.164 | 0.358 | 0.464 | 0.253 | 0.035 | 0.472 | 0.023* |
|  | BMI | 0.011 | -0.028 | 0.050 | 0.586 | 0.082 | 0.036 | 0.127 | 0.001* |
|  | Alcohol | 0.081 | -0.169 | 0.330 | 0.525 | -0.156 | -0.405 | 0.094 | 0.220 |
|  | Smoking | 0.150 | -0.113 | 0.412 | 0.263 | 0.129 | -0.128 | 0.386 | 0.324 |
|  | APOE2+ | -0.356 | -0.689 | -0.022 | 0.037* | -0.279 | -0.615 | 0.058 | 0.104 |
|  | APOE4+ | 0.084 | -0.198 | 0.367 | 0.557 | -0.067 | -0.344 | 0.210 | 0.634 |
| HDL-C | Constant | 0.140 | -0.911 | 1.190 | 0.794 | 1.337 | 0.847 | 1.827 | 0.000 |
|  | Age | 0.008 | 0.002 | 0.014 | 0.006* | -0.003 | -0.006 | -0.001 | 0.012* |
|  | Sex | 0.177 | -0.042 | 0.396 | 0.112 | 0.055 | -0.018 | 0.127 | 0.139 |
|  | BMI | 0.030 | -0.002 | 0.063 | 0.069 | 0.000 | -0.016 | 0.015 | 0.961 |
|  | Alcohol | 0.209 | 0.000 | 0.418 | 0.050 | -0.057 | -0.139 | 0.026 | 0.180 |
|  | Smoking | -0.202 | -0.422 | 0.018 | 0.072 | 0.000 | -0.086 | 0.085 | 0.991 |
|  | APOE2+ | -0.046 | -0.325 | 0.234 | 0.748 | -0.059 | -0.171 | 0.053 | 0.299 |
|  | APOE4+ | 0.003 | -0.233 | 0.240 | 0.978 | -0.034 | -0.126 | 0.058 | 0.465 |
| LDL-C | Constant | 2.026 | 1.030 | 3.021 | 0.000 | 0.570 | -0.677 | 1.817 | 0.369 |
|  | Age | 0.002 | -0.003 | 0.007 | 0.459 | 0.007 | 0.001 | 0.013 | 0.048* |
|  | Sex | 0.190 | -0.017 | 0.398 | 0.072 | 0.299 | 0.114 | 0.483 | 0.002* |
|  | BMI | 0.000 | -0.031 | 0.031 | 0.989 | 0.055 | 0.016 | 0.093 | 0.006* |
|  | Alcohol | 0.026 | -0.172 | 0.224 | 0.797 | 0.029 | -0.182 | 0.239 | 0.790 |
|  | Smoking | 0.179 | -0.029 | 0.388 | 0.092 | 0.222 | 0.005 | 0.440 | 0.045* |
|  | APOE2+ | -0.256 | -0.521 | 0.008 | 0.058 | -0.176 | -0.460 | 0.109 | 0.225 |
|  | APOE4+ | 0.016 | -0.209 | 0.240 | 0.891 | -0.103 | -0.337 | 0.132 | 0.389 |
| TG | Constant | 0.582 | -1.349 | 2.513 | 0.554 | 1.032 | -0.938 | 3.001 | 0.303 |
|  | Age | -0.004 | -0.014 | 0.007 | 0.460 | 0.000 | -0.010 | 0.011 | 0.958 |
|  | Sex | -0.322 | -0.724 | 0.081 | 0.117 | -0.261 | -0.553 | 0.031 | 0.079 |
|  | BMI | 0.104 | 0.044 | 0.164 | 0.001* | 0.067 | 0.006 | 0.129 | 0.031* |
|  | Alcohol | -0.326 | -0.711 | 0.058 | 0.096 | -0.227 | -0.560 | 0.106 | 0.181 |
|  | Smoking | 0.076 | -0.328 | 0.481 | 0.711 | -0.067 | -0.410 | 0.276 | 0.701 |
|  | APOE2+ | 0.223 | -0.291 | 0.737 | 0.394 | -0.080 | -0.529 | 0.369 | 0.726 |
|  | APOE4+ | 0.024 | -0.411 | 0.459 | 0.914 | 0.136 | -0.234 | 0.507 | 0.469 |
| C-index 1 | Constant | 3.720 | 1.662 | 5.778 | 0.000 | 1.437 | -2.874 | 5.749 | 0.512 |
|  | Age | -0.010 | -0.021 | 0.001 | 0.081 | 0.014 | -0.008 | 0.037 | 0.209 |
|  | Sex | -0.385 | -0.813 | 0.044 | 0.079 | 0.070 | -0.569 | 0.709 | 0.829 |
|  | BMI | 0.026 | -0.038 | 0.090 | 0.428 | 0.110 | -0.024 | 0.244 | 0.106 |
|  | Alcohol | -0.191 | -0.601 | 0.219 | 0.359 | -0.338 | -1.067 | 0.391 | 0.362 |
|  | Smoking | 0.656 | 0.225 | 1.087 | 0.003* | 0.151 | -0.601 | 0.903 | 0.693 |
|  | APOE2+ | -0.043 | -0.590 | 0.505 | 0.879 | -0.106 | -1.089 | 0.877 | 0.831 |
|  | APOE4+ | 0.007 | -0.457 | 0.470 | 0.977 | 0.888 | 0.077 | 1.698 | 0.032* |
| C-index 2 | Constant | 1.698 | 0.273 | 3.123 | 0.020 | -0.048 | -2.407 | 2.311 | 0.968 |
|  | Age | -0.007 | -0.015 | 0.001 | 0.078 | 0.013 | 0.001 | 0.025 | 0.038* |
|  | Sex | -0.068 | -0.365 | 0.228 | 0.650 | 0.192 | -0.158 | 0.541 | 0.282 |
|  | BMI | 0.017 | -0.027 | 0.061 | 0.448 | 0.067 | -0.006 | 0.140 | 0.073 |
|  | Alcohol | 0.056 | -0.228 | 0.340 | 0.699 | -0.043 | -0.442 | 0.356 | 0.833 |
|  | Smoking | 0.378 | 0.079 | 0.676 | 0.013* | 0.213 | -0.198 | 0.624 | 0.309 |
|  | APOE2+ | -0.114 | -0.493 | 0.265 | 0.554 | -0.085 | -0.623 | 0.453 | 0.756 |
|  | APOE4+ | -0.042 | -0.363 | 0.279 | 0.798 | 0.420 | -0.024 | 0.863 | 0.064 |

Note: Unstandardized coefficients and P-values were acquired by using linear regression model. *Statistically Significant.
